# Association between thyroid dysfunction and type 2 diabetes: a meta-analysis of prospective observational studies

**DOI:** 10.1186/s12916-021-02121-2

**Published:** 2021-10-21

**Authors:** Fen Rong, Hongji Dai, Yuwan Wu, Jibin Li, Guoping Liu, Hanbei Chen, Xi Zhang

**Affiliations:** 1 School of Public Health, Shanghai University of Traditional Chinese Medicine, Shanghai, China; 2 Department of Epidemiology and Biostatistics, National Clinical Research Center for Cancer, Key Laboratory of Molecular Cancer Epidemiology of Tianjin, Tianjin Medical University Cancer Institute and Hospital, Tianjin Medical University, Tianjin, China; 3 Department of Pediatrics, Xin Hua Hospital, Shanghai Jiao Tong University School of Medicine, Shanghai, China; 4 Department of Clinical Research, Sun Yat-sen University Cancer Center, State Key Laboratory of Oncology in Southern China, Collaborative Innovation Center for Cancer Medicine, Guangzhou, China; 5 School of Basic Medical Sciences, Shanghai University of Traditional Chinese Medicine, 201203 Shanghai, China; 6 Department of Endocrinology, Xin Hua Hospital, Shanghai Jiao Tong University School of Medicine, No. 1665 Kongjiang Road, Shanghai, 200092 China; 7 Clinical Research Unit, Xin Hua Hospital, Shanghai Jiao Tong University School of Medicine, No. 1665 Kongjiang Road, Shanghai, 200092 China

**Keywords:** Thyroid dysfunction, Type 2 diabetes, Meta-analysis, Prospective study

## Abstract

**Background:**

Diabetes mellitus and thyroid disease are two areas of broad interest in the field of endocrinology and metabolism. Variation of thyroid hormone concentration, even within the normal range, may portend the onset of type 2 diabetes mellitus (T2DM), especially among those with prediabetes. However, current evidence is mixed.

**Methods:**

Prospective studies which assessed diabetes incidence were identified using a database search of MEDLINE and Embase through May 1, 2021. The Sidik-Jonkman random-effects model and cubic spline model were used to evaluate the associations and dose-response relationships between thyroid function/hormone levels and risk of T2DM and cardiovascular disease (CVD) risk among T2DM patients.

**Results:**

A total of 12 prospective studies were included. We found that high baseline TSH levels were related to a 17% higher risk of T2DM (RR 1.17, 95% CI 1.01, 1.36; *I*^2^=78%, *P*<0.01), compared with normal TSH levels. Low FT3 (RR 1.40, 95% CI 1.09, 1.80; *I*^2^=59%, *P*=0.03) and low FT4 (RR 1.33, 95% CI 1.04, 1.71; *I*^2^=62%, *P*=0.02) levels were significantly associated with risk of T2DM. The cubic spline model indicated a J-shaped relationship with TSH, but inverted-J-shaped relationships with FT3 and FT4. CVD events and all-cause deaths were prospectively evaluated in four studies, but were not associated with abnormal thyroid function.

**Conclusions:**

Our meta-analysis determined that abnormal thyroid hormone level is associated with an increased risk of T2DM, showing a J-shaped relationship with TSH and inverted-J-shaped relationships with FT3 and FT4.

**Trial registration:**

Registered number in PROSPERO: CRD42021225695.

**Supplementary Information:**

The online version contains supplementary material available at 10.1186/s12916-021-02121-2.

## Background

Type 2 diabetes mellitus (T2DM) is a chronic metabolic disease that results from pancreatic beta-cell dysfunction and peripheral insulin resistance. The worldwide prevalence is 9.1%, equating to 415 million adults suffering from the disease [1]. T2DM is a complex disease arising from a combination of genetic and lifestyle factors. Recently, evidence has suggested that low circulating levels of thyroid hormone, even within the normal reference concentrations, may be related to an elevated risk of developing T2DM, especially within the prediabetes population [2].

Like diabetes, thyroid dysfunction results from dysregulated hormone secretion. Recent data from the Colorado Thyroid Disease Prevalence study showed that 9.5% of 25,862 participants had an elevated thyroid-stimulating hormone (TSH); conversely, 2.2% had low TSH [3]. The thyroid hormone axis includes TSH, thyroxine (T4), and triiodothyronine (T3), all of which are required to maintain the normal functioning of the thyroid. The imbalance of these hormones may lead to metabolic overactivity (hyperthyroidism; excess thyroid hormone) or underactivity (hypothyroidism; inadequate thyroid hormone). Based on the severity of the imbalance, hyperthyroidism and hypothyroidism can be diagnosed as either clinical or subclinical, of which the latter is the more prevalent [4]. Subclinical thyroid dysfunction is commonly defined as having an abnormal TSH but normal T4 concentration. The presence or absence of symptoms may be independent of T4 levels [5, 6]. Despite being mild, subclinical thyroid dysfunction has been linked to several complications, including cardiovascular disease (CVD) [7], chronic kidney disease [8], and type 1 diabetes in children [9].

Thyroid dysfunction has been reported to be associated with T2DM in a number of studies [10–12]. Some studies have suggested a bidirectional influence of diabetes and thyroid disorders upon each other [10, 13]. The Third National Health and Nutrition Examination Survey (NHANES III), a large cross-sectional survey study which included 17,353 participants in the USA, revealed that hypothyroidism was present in 4.6% of the study population and hyperthyroidism in 1.3% of subjects [14]. In addition, NHANES III found an increased frequency of thyroid dysfunction in subjects with diabetes compared to those without diabetes.

Thyroid hormone has been demonstrated to regulate carbohydrate metabolism and pancreatic function [15]. Conversely, diabetes can variably influence thyroid function. For example, the response of TSH to thyrotropin-releasing hormone has been shown to be impaired in diabetes, leading to hypothyroidism and concomitant lower T3 levels [16]. It has been suggested that lower T3 levels may also be explained by a lower level of conversion of T3 from T4 in diabetes based on studies of hyperglycemia-induced reversible reduction to deiodinase activity and hepatic concentration of thyroxine [17]. Other studies have suggested that short-term T3 excess may induce insulin resistance; hence leading to T2DM [18, 19].

However, the relationship between thyroid hormone levels and T2DM risk remains highly contested and human studies have demonstrated conflicting findings. Several reports have suggested a positive effect of high TSH and low free thyroxine levels on hyperglycemia and insulin resistance [20–23], but some claimed no relationship found in their researches [24]. Therefore, it has become apparent that a comprehensive evaluation of the association between TSH, free thyroxine, and T2DM is needed. Moreover, virtually all previous analyses have focused on examining the influence of baseline levels of TSH and free thyroxine on the risk of developing T2DM [25–27], but few studies have investigated the dose-response influence of thyroid hormone levels on T2DM risk, so this analysis was conducted here.

## Methods

### Data sources and searches

All data sources were obtained from the EMBASE and MEDLINE libraries. A systematic search was performed for publications before May 1, 2021, including prospective studies on the association between thyroid function (hormone levels), risk of T2DM, and prospective studies of linkage between thyroid function, CVD, and CVD-related outcomes among T2DM subjects. Keywords included “diabetes,” “type 2 diabetes,” or “T2DM” for T2DM and “subclinical thyroid dysfunction,” “thyroid function,” “hyperthyroidism,” “hypothyroidism” “thyroid-stimulating hormone,” “free thyroxine,” “T3,” or “T4.” The Boolean logical operator AND was used to combine the diabetes and thyroid function terms, and operator OR between terms within those categories. All searches were limited to English language and adults. Additionally, bibliographies of related articles and current review articles were manually screened for potentially relevant articles. The detailed electronic search strategy is shown in the supplementary file.

### Study selection

The Preferred Reporting Items for Systematic Reviews and Meta-Analyses (PRISMA) statement was used to report systematic reviews included in this analysis [28]. Prospective observational studies that evaluated the associations between thyroid function (or thyroid hormone) and risk of T2DM were included. Studies that assessed the influence of thyroid function (or hormone levels) on CVD and CVD-related outcomes among T2DM subjects were also included. In order to exclude publications, the following exclusion criteria were used: (1) not original studies (reviews, meta-analysis, meeting abstracts, editorials, letters, or commentaries); (2) studies which included populations aged less than 18 years or pregnant women; (3) non-prospective study design, including cross-sectional, case-control, or retrospective cohort studies; and (4) studies including the patients with malignancy, severe infectious disease, studies without reporting the odds ratio (OR) or hazard ratio (HR) of the baseline thyroid function or thyroid hormone levels for the risks of T2DM or CVD/CVD-related events among T2DM patients.

For all search results, duplicated articles were removed and a title and abstract review was conducted to exclude obviously irrelevant articles. The remaining articles were screened using a full-text review based on the above inclusion and exclusion criteria to identify eligible articles. Two independent investigators (Y.W. and X.Z.) examined each eligible article and were responsible for determining which publications were included in the final analysis. Any discrepancies would be discussed with a third investigator (F.R.).

### Data extraction and quality assessment

Y.W. extracted data along with relevant information from all selected articles and entered it into a standard form. The content included general information of the articles (first author’s name, year published, and country), participants (number of participants and T2DM/CVD/CVD-related cases, sex percentage, race percentage, age range, mean/median/range of BMI, and thyroid medication use), study design (follow-up years, outcomes, adjusted variables, and comparisons). If two articles derived from the same study population both reported HRs/ORs, the one with a larger sample size was selected. The accuracy of the data and information was confirmed by another researcher (F.R.).

The Newcastle-Ottawa Scale (NOS) was applied to assess the quality of identified prospective studies [29]. Three components of the NOS scale, selection, comparability, and outcome were used to rate the studies as low quality (≤ 6 stars) or high quality (> 6 stars).

### Data synthesis and analysis

We analyzed the studies for thyroid function/hormone levels and risk of T2DM and the studies relating to the CVD risk with thyroid function among T2DM patients. We estimated the pooled relative risk (RRs) and their corresponding 95% confidence interval (CIs) using the Sidik-Jonkman random-effects model [30]. The between-study heterogeneity was assessed by Cochran’s *Q* statistic with values of *P* < 0.10 used as the indicator of a statistically significant result. The proportion of heterogeneity was evaluated by the *I*^2^ index (*I*^2^ > 25% indicates medium or high heterogeneity).

In order to identify the major source of heterogeneity and evaluate the robustness of pooled results, a prespecified subgroup analysis was performed. The subgroup factors included characteristics of study participants including the age of participants (< 60 years, ≥ 60 years, not reported), sex (women < 50%, women ≥ 50%, not reported), BMI (normal weight, overweight or obese, not reported), and thyroid medication (thyroid replacement or antithyroid medication) use (no, mixed, not reported); characteristics of study design: study location (Asia, Europe, & the USA, not reported), sample size (≤ median, > median, not reported), and follow-up years (≤ 5 years, > 5 years, not reported); and quality of studies according to the NOS (6, 7, and 8 stars). Publication bias of the included studies was visually explored by funnel plots and examined by Begger’s adjusted rank correlation tests [31].

Additionally, a dose-response meta-analysis was performed in order to evaluate whether there was a dose-response relationship between thyroid function levels, including TSH, FT3, and FT4 concentration, in relation to T2DM. Cubic spline regression models were applied to fit the relation-shapes of thyroid function levels and T2DM using 3 fixed knots at 10%, 50%, and 90% percentiles.

A sensitivity analysis was conducted to evaluate whether results from the individual study could substantially influence the pooled results of thyroid function levels and T2DM.

All analyses were conducted using the R software package (version 3.6.0, R Foundation for Statistical Computing, Vienna, Austria) and Stata (version 14; StataCorp, College Station, TX). A two-tailed *P* < 0.05 was considered statistically significant.

## Results

**Fig. 1:**
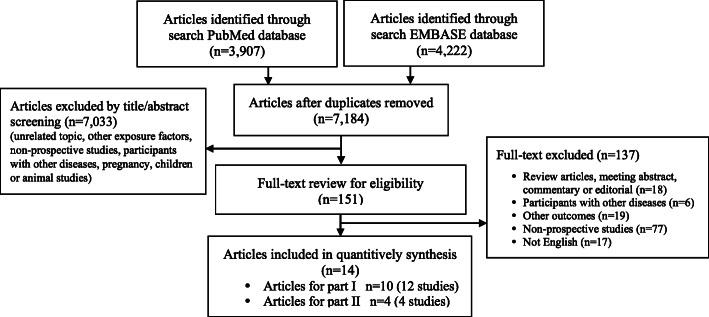
Flow chart for article selection

Using both electronic and manual searches, a total of 7184 articles were identified after the removal of 945 duplicates. After title/abstract screening, 151 articles were included for further full-text review. Of those, a total of 14 articles were identified for inclusion, among them, 10 articles (12 studies) for analyzing the association between thyroid status and T2DM risk, and an additional 4 articles (4 studies) for evaluating the association between thyroid status and CVD prognosis among T2DM patients (Fig. 1).

### Characteristics of included prospective studies

We identified a total of 12 prospective studies from 8 countries in the final analysis of the association between thyroid function levels and risk of T2DM, which comprised 17,828 T2DM cases among 337,823 participants during a median follow-up of 6 years. All these identified studies were large-scale studies with a sample size ranging from 2316 to 91,120 and were high-quality studies with a NOS quality scale range of 6–8 stars. Most of the studies (*n*=10) focused on the incidence of DM or T2DM, whereas one study evaluated diabetic mortality, and one study measured fasting blood glucose levels. A summary of the included studies is presented in Table 1.

**Table 1:** Study characteristics of 16 prospective observational studies included in the meta-analysis

Author, publication year	Cohort, country	Design, follow-up years	Population, cases/total no. of subjects	Age (years), female (%), and ethnicity	BMI (kg/m^**2**^), thyroid medicine users (%)	Outcomes	Covariates adjusted in models
M Thvilum, 2013 [32]	Official Danish health registers study, Denmark	Prospective cohort study, mean: 6 years	Danish national patient registry population, 1020/14,480	Mean: 58, 84% of women, NR	NR, no	T2DM	Age and sex.
N Gronich, 2015 [2]	NR, Israel	Prospective cohort study, 5–7 years	Statin non-users 3666/20,334	40–80, 62.11% of women, NR	Obesity (43.63%), mixed	DM	Age, sex, race, obesity, smoking, glucose, LDL, HDL, TG, history of hypertension, and drug use.
N Gronich, 2015 [2]	NR, Israel	Prospective cohort study, 5–7 years	Statin users, 4410/39,263	40–80, 62.24% of women, NR	Obesity (44.3%), mixed	DM	Age, sex, race, obesity, smoking, glucose, LDL, HDL, TG, history of hypertension, and drug use.
J-E Jun, 2016 [33]	NR, Korea	Prospective cohort study, 7 years	Population-based, 229/6235	≥ 18, 41.96% of women, NR	Mean (SD): 23.7 (2.56), no	T2DM	Age, sex, BMI, HbA1c, HDL, TG, LDL, smoking, lipid drug use, hypertension, BMI change.
L Mehran, 2016 [34]	Tehran Thyroid Study, Iran	Prospective cohort study, median: 9.73 years	Population-based, NR/2316	≥ 20, NR, NR	Mean (SD): 25.3 (4.3), no	High-fasting blood glucose	Age, sex, smoking, BMI, and HOMA-IR.
L Chaker, 2016 [35]	The Rotterdam Study, Netherlands	Prospective cohort study, mean: 7.9 years	Healthy population-based, 757/8107	Mean: 64.6, 58% of women, NR	Mean (SD): 26.5 (4.05), mixed	DM	Sex, age, smoking, cohort, FSG levels, insulin, SBP, DBP, antihypertensive drugs, HDL cholesterol, and BMI.
C-H Chang, 2017 [27]	NR, China	Prospective cohort study, median: 2.6 years	Population-based, 1551/68,846	Mean: 41.2, 52.5% of women, NR	Obesity (3.2%), no	Dysglycemia, prediabetes, and T2DM	Sex, age, education level, smoking, drinking, and obesity.
N M.Y. Journy, 2017 [36]	U.S. radiologic technologist cohort, USA	Prospective cohort study, median: 28 years	Women, 163/75,076	20-89, 100% of women, multiple	NR, mixed	T2DM deaths	Baseline year, age, race, BMI, family history of breast cancer, lifestyle, reproductive factors.
T. Ittermann, 2017^a^ [37]	Study of Health in Pomerania, Germany	Prospective cohort study, median: 5 years	Population-based, 116/2689	20–79, 51.2% of women, NR	Mean (SD): 27.2 (4.7), mixed	T2DM	Age and sex.
T. Ittermann, 2017^a^ [37]	INTER99, Denmark	Secondary analysis of RCT, 5 years	Population-based, 142/3815	30–60, 49.9% of women, NR	Mean (SD): 26.3 (4.6), NR	T2DM	Age and sex.
T. I. de Vries, 2018 [38]	SMART study, Netherlands	Prospective cohort study, 2.9–8.3 years	With high cardiovascular risk, 289/5542	Mean: 56, 35.24% of women, NR	Mean (SD): 26.72 (4), no	T2DM	Age, sex, current smoking, total and HDL cholesterol, and triglycerides
R-H Chen, 2019 [39]	NR, China	Prospective cohort study, 10 years	Population-based, 5485/91,120	≥ 18, 77.4% of women, NR	Obesity (0.7%), NR	T2DM	Age, sex, and comorbidities.
H-S Chen, 2007 [40]	NR, China	Prospective cohort study, 3–4.3 years	T2DM patients, CVD: 61, CVD deaths: 12, deaths: 31/556	≥ 30, 35.07% of women, NR	Mean (SD): 26.11 (9.8), mixed	CVD events, CVD deaths, and all-cause deaths	Age, sex, HbA1c, TC, HDL, BP, BMI, smoking, medication, urinary albumin, creatinine
C Drechsler, 2014 [41]	NR, China	Prospective cohort study, 4 years	Diabetic hemodialysis patients, sudden death: 120 MI: 156, stroke: 70, CV events: 345, deaths: 441/ 934	18-80, 46.26% of women, NR	Mean (SD): 27.6 (4.98), NR	Sudden death, MI, stroke, combined CV events, all-cause death	Age, sex, atorvastatin, SBP, BMI, left ventricular hypertrophy, albumin, creatinine, N-terminal pro2B-type natriuretic peptide, ultrafiltration volume.
J Geng, 2014 [42]	NR, China	Prospective cohort study, 2–3 years	T2DM patients, CHD: 32, new AF: 14, deaths: 10/694	Mean: 56.1, 32.75% of women, NR	Mean (SD): 25.13 (3.63), NR	CHD events, new-AF, and all-cause deaths	Age, sex, hypertension, total cholesterol, LDL-cholesterol, smoking, SBP, DBP, and BMI.
T I. de Vries, 2019 [26]	SMART study, Netherlands	Prospective cohort study, 3.3–9.6 years	T2DM patients, CVD: 191, deaths: 204/1265	Mean 61, 26.64% of women, NR	Mean (SD): 29.22 (5), NR	CVD, all-cause deaths	Age, sex, smoking, history of vascular disease, eGFR, SBP, TC, and HDL-C.

*Abbreviations*: *NR* not reported, *BMI* body mass index, *DM* diabetes mellitus, *T2DM* type 2 diabetes mellitus, *LDL* low-density lipoprotein, *HDL* high-density lipoprotein, *TG* triacylglycerol, *TC* cholesterol, *AF* atrial fibrillation, *BP* blood pressure, *SBP* systolic blood pressure, *DBP* diastolic blood pressure, *CVD* cardiovascular disease, *MI* myocardial infarction, *CHD* coronary heart disease, *FSG* fasting serum glucose, *eGFR* estimated glomerular filtration rate

^a^These two studies were extracted from one article

Additionally, 4 studies which prospectively evaluated for CVD were identified, 3 studies from China, and another 1 from the Netherlands. These studies included a total of 3449 T2DM patients with a follow-up of 2.0–9.6 years. The sample sizes ranged from 556 to 1265 and the NOS scale of all studies was 6 stars.

### Thyroid function levels and risk of T2DM

**Fig. 2:**
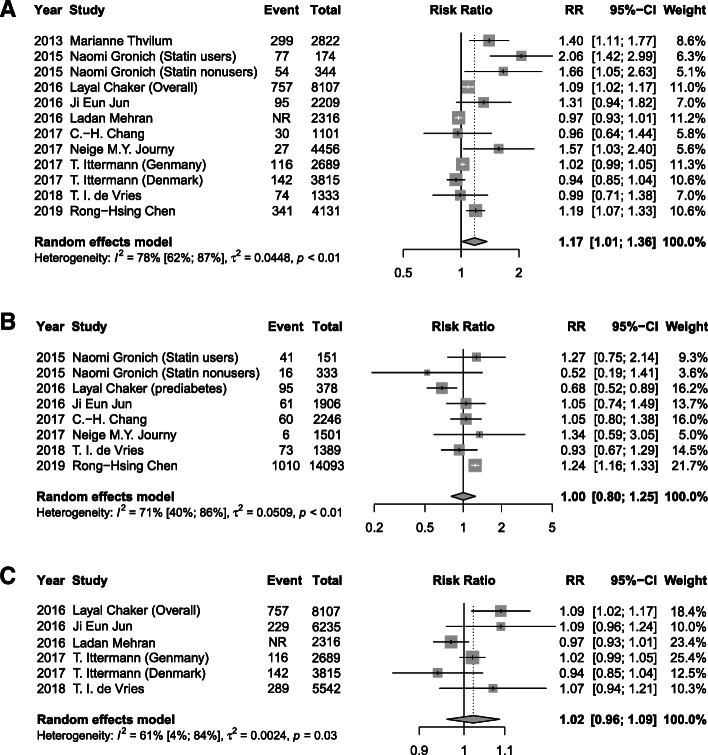
Meta-analysis for associations between serum TSH levels, including high TSH levels: >2.21 mIU/L (**A**), low TSH levels < 1.5 mIU/L (**B**), and TSH levels as continuous variable with a median of 1.95 mIU/L (**C**) and risk of T2DM

**Fig. 3:**
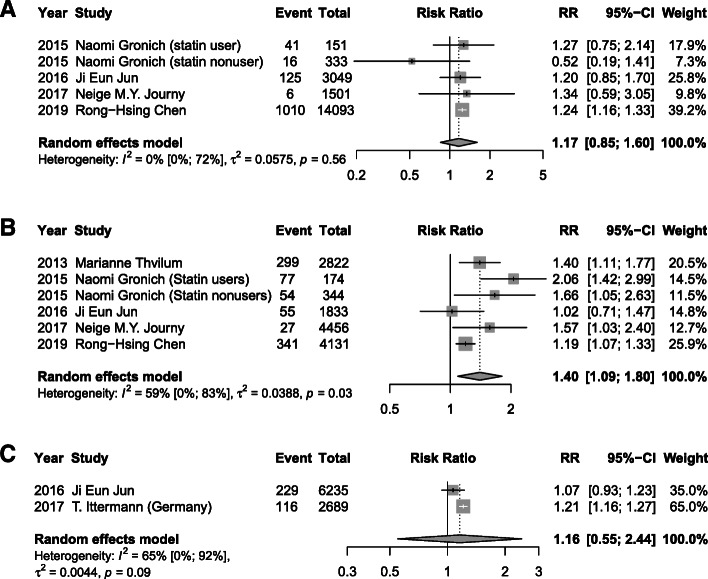
Meta-analysis for associations between serum FT3 levels, including high FT3 levels: > 7.1 pmol/L (**A**), low FT3 levels < 2.8 pmol/L (**B**), and FT3 levels as continuous variable with a median of 4.77 pmol/L (**C**), and risk of T2DM

**Fig. 4:**
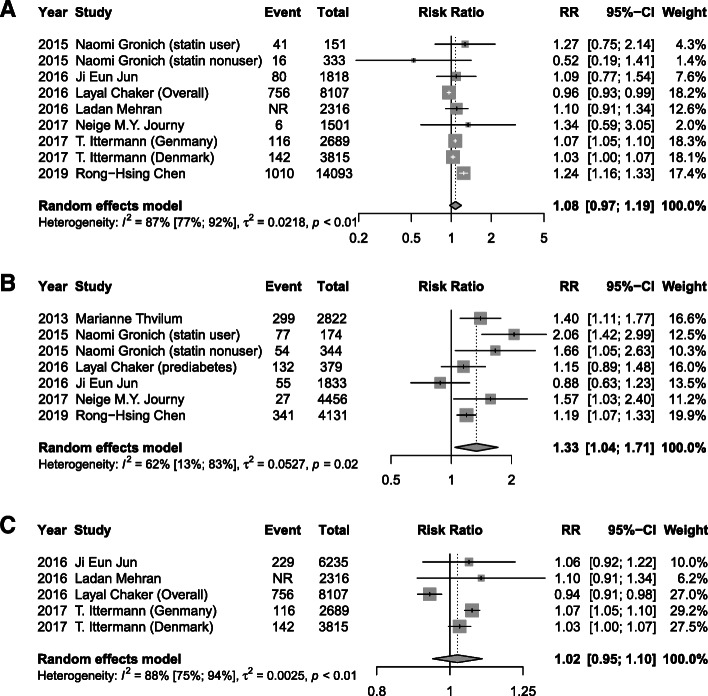
Meta-analysis for associations between serum FT4 levels, including high FT4 levels: > 27.65 pmol/L (**A**), low FT4 levels < 15.32 pmol/L (**B**), and FT4 levels as continuous variable with a median of 17.18 pmol/L (**C**) and risk of T2DM

Meta-analysis of 12 studies demonstrated that a high baseline TSH level was associated with a 17% higher risk of T2DM (RR 1.17, 95% CI 1.01, 1.36; *I*^2^=78%, *P* < 0.01) after a median of 6 years follow-up, when compared with a normal TSH (Fig. 2A). A significant association of low serum FT3 concentrations (RR 1.40, 95% CI 1.09, 1.8; *I*^2^=59%, *P* = 0.03) and low FT4 (RR 1.33, 95% CI 1.04, 1.71; *I*^2^=62%, *P* = 0.02) with incidence of T2DM were also found (Figs. 3B and 4B). However, low TSH levels (RR 1.00, 95% CI 0.80, 1.25; *I*^2^=71%), high FT3 (RR 1.17, 95% CI 0.85, 1.60; *I*^2^=0%), and high FT4 (RR 1.08, 95% CI 0.97, 1.19; *I*^2^=87%) were found to not be significantly related with T2DM risk (Figs. 2B, 3A, and 4A). Additionally, as continuous variables, TSH (RR 1.02, 95% CI 0.96, 1.09; *I*^2^=61%), FT3 (RR 1.16, 95% CI 0.55, 2.44; *I*^2^=65%), or FT4 (RR 1.02, 95% CI 0.95, 1.10; *I*^2^=88%) did not significantly correlate with the risk of T2DM (Figs. 2C, 3C, and 4C). No substantial publication bias was detected for any of the above analyses using Begger’s tests.

### Dose-response association between thyroid function levels and T2DM risk

**Fig. 5:**
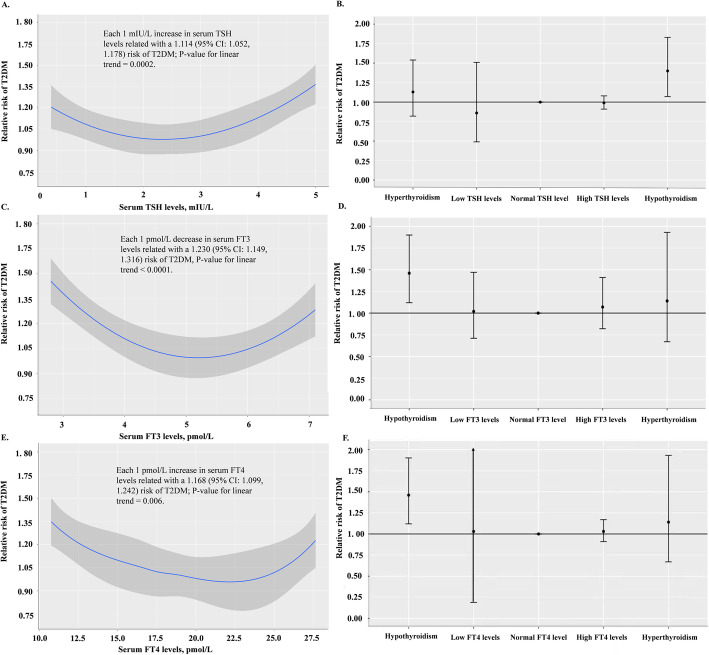
Dose-response relationship between serum TSH levels and T2DM risk (**A**, **B**), serum FT3 levels and T2DM risk (**C**, **D**), and serum FT4 levels and T2DM risk (**E **, **F**). The nonlinear relation was fitted using a restricted cubic spline regression curve among 9 pr ospective studies

In order to further evaluate the dose-response relationship between thyroid function levels and risk of T2DM, 9 studies were analyzed with a regression model. A positive linear relationship between TSH levels and T2DM risk was found. Serum TSH levels above 5.0 mIU/L, the upper limit of normal, conferred a higher risk of T2DM, while each 1 mIU/L increase in serum TSH corresponded to an 11.4% elevation of the risk for T2DM (95% CI 1.052, 1.178) (Fig. 5A, B). A negative linear relationship was found between both FT3 and FT4 levels at T2DM risk. Lower serum FT3/FT4 levels than 2.8/10.75 pmol/L, the lowest concentrations of FT3 and FT4, respectively, were associated with a high risk of T2DM, and each 1 pmol/L decrease in serum FT3/FT4 related with 23.0% (95% CI 1.149, 1.316) or 16.8% (95% CI 1.099, 1.242) elevation of the T2DM risk, respectively (Fig. 5C-F). After adjustment for variables related to thyroid function, the associations remained consistent and significant.

### Subgroup and sensitivity analyses

Due to the limited number of studies included, subgroup analyses on the TSH-T2DM association stratified by age (age < 60 years and ≥ 60 years), sex (women ≥ 50% and women < 50%), study location (Asia and Europe/USA), BMI (normal and overweight/obese), follow-up years (≤ 5 years and > 5 years), thyroid medication users (no, mixed, and not reported), sample size (≤ median and > median), and quality score (6, 7, and 8 stars) were applied. A significant association between high levels of TSH and T2DM was found among studies of participants with normal weight and follow-up years longer than 5 years, but not in those who were overweight or obese or with follow-up years less or equal to 5 years (all *P* values for interaction < 0.05) (Table 2). Additionally, studies which did not report the information on thyroid medication use tended to have a significant association between low levels of TSH and high risk of T2DM, but not in those including both users and non-users [27, 36] (*P* value for interaction = 0.007).

**Table 2:** Stratified meta-analysis of associations between high TSH and low TSH levels with T2DM risk

Subgroups	High TSH levels (> 2.21 mIU/L)			Low TSH levels (< 1.5 mIU/L)		
	No. of studies	RR (95% CI)	***P*** value	No. of studies	RR (95% CI)	***P*** value
**Age,** **years**^**a**^						
**< 60**	9	1.104 (0.965; 1.263)	0.182	5	1.113 (0.945; 1.309)	0.299
**≥ 60**	4	1.376 (1.025; 1.847)		4	0.897 (0.618; 1.302)	
**Sex**						
**Women (≥ 50%)**	9	1.252 (1.058; 1.483)	0.065	6	1.003 (0.752; 1.337)	0.521
**Women (< 50%)**	4	0.978 (0.799; 1.197)		3	1.120 (0.938; 1.337)	
**Study location **						
**Asia**	6	1.264 (0.997; 1.604)	0.377	5	1.099 (0.852; 1.417)	0.251
**Europe & the USA**	6	1.113 (0.955; 1.298)		3	0.851 (0.596; 1.215)	
**BMI, kg/m**^**2a**^						
**Normal**	2	1.404 (1.072; 1.840)	**0.005**	2	1.093 (0.780; 1.533)	0.238
**Overweight/obese**	5	1.009 (0.945; 1.077)		2	0.784 (0.579; 1.062)	
**Not Reported**	5	1.381 (1.074; 1.777)		4	1.096 (0.785; 1.530)	
**Follow-up years**						
**≤ 5** **years**	4	1.004 (0.912; 1.105)	**0.024**	2	1.141 (0.955; 1.363)	0.251
**> 5** **years**	9	1.263 (1.060; 1.505)		7	0.959 (0.757; 1.216)	
**Thyroid medicine users **						
**No**	9	1.204 (1.002; 1.447)	0.617	4	0.899 (0.773; 1.045)	** 0.007**
**Mixed (yes & no)**	4	1.084 (0.900; 1.306)		2	1.076 (0.828; 1.398)	
**Not Reported**	2	1.057 (0.846; 1.321)		3	1.236 (1.155; 1.322)	
**Sample size**						
**≤ median **	6	1.234 (0.953; 1.599)	0.587	3	0.844 (0.592; 1.202)	0.071
**> median**	6	1.138 (0.989; 1.309)		5	1.190 (1.058; 1.340)	
**Quality score **						
**6 stars**	4	1.127 (0.940; 1.350)	0.240	3	1.156 (0.942; 1.419)	0.405
**7 stars**	5	1.075 (0.908; 1.272)		2	1.050 (0.845; 1.304)	
**8 stars**	3	1.502 (1.057; 2.134)		3	0.806 (0.490; 1.326)	
**Outcomes**						
**T2DM**	5	1.059 (0.944; 1.188)	0.127	4	0.970 (0 .750; 1.256)	0.722
**T2DM & T1DM**	7	1.297 (1.048; 1.606)		4	1.057 (0.7 11; 1 .574)	

^a^Age and BMI categories were divided according to the means of age and BMI levels reported by studies

### Thyroid fu nction levels and CVD and all-cause death risks among T2DM patients

**Fig. 6:**
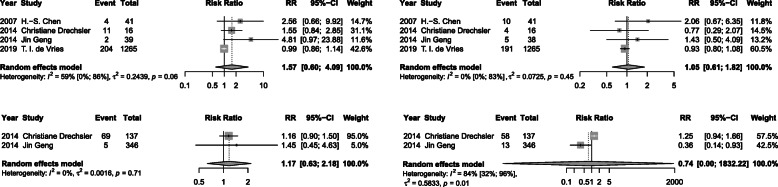
Meta-analysis for associations of serum TSH levels (high TSH levels and low TSH levels) with CVD events and all-cause deaths among T2DM patients

Meta-analysis of 4 studies which reported CVD outcomes revealed that neither high nor low TSH levels were associated with either CVD risk or all-cause mortality risk (Fig. 6). A relatively high heterogeneity between the 2 studies included for low TSH and CVD risk was detected (*I*^2^=84%, *P* = 0.01). No substantial publication bias was detected for any of the above analyses using Begger’s tests (P_Begger_ > 0.05).

## Discussion

The goal of our meta-analysis was to evaluate the relationship between thyroid function and T2DM. We found that high TSH and low FT3/FT4 are associated with a higher risk of developing T2DM and that the risk of T2DM rises in a dose-dependent manner as TSH rises and thyroid hormones decrease. Investigations determined that for each 1 mIU/L elevation in TSH, 1 pmol/L reduction in FT3 or FT4 was associated with either an 11%, 23%, and 16.8% higher risk for T2DM among the normal population, respectively. Thyroid function tests were not associated with CVD or all-cause mortality among T2DM patients; this may be due to the limited number of studies, and additional studies in this area are warranted.

Our results confirmed that hypothyroidism seems to be associated with an elevated risk T2DM. A previous review examined the association between subclinical hypothyroidism and diabetic complications [43] in T2DM. This meta-analysis on 36 case-control and cross-sectional studies found that T2DM patients were more likely to have subclinical hypothyroidism when compared to the general population, and T2DM patients with subclinical hypothyroidism were more likely to have complications (such as diabetic nephropathy, retinopathy, and peripheral neuropathy). Numerous studies have suggested that the diabetes-thyroid relationship might be bidirectional [44]. High insulin levels found in prediabetes and early type 2 diabetes can stimulate thyroid tissue hyperplasia, leading to thyroid enlargement and nodule formation [45, 46]. Thyroid dysfunction may in turn affect the glucose metabolism in diabetes. Changes in serum TSH were found to be correlated with changes in glycated hemoglobin (HbA1c) [47]. It is reported that improvement of glycemic control was significantly related with a reduction of major cardiovascular events [48]. Since hyperthyroidism may worsen the glucose control in diabetic patients, while hypothyroidism can increase the risk of hypoglycaemia, and therefore, both may increase the risk of cardiovascular disease [49–51]. Nevertheless, we did not observe significant associations of low TSH, high FT3, or high FT4 with risk of T2DM. This could be partially explained by their “J-shaped” or “inverted J-shaped” relationship, indicating a weaker association with low TSH, high FT3, and high FT4, compared with high TSH, low FT3, and low FT4, respectively. Hyper- and hypothyroidism have different underlying mechanism to impact the glucose homeostasis. Hyperthyroidism could promote hyperglycaemia [52] and reduce the half-life of insulin, leading to an increased rate of degradation and an enhanced release of biologically inactive insulin precursors [18, 53]. As for hypothyroidism, glucose metabolism is affected via a reduced rate of liver glucose production [54] and the decrease in insulin requirement. The cross-sectional study observed a much higher prevalence of hypothyroidism (~30%) than hyperthyroidism (12%) in diabetes [55]. However, limited directly prospective evidence on which one, hypothyroidism vs. hyperthyroidism, is more likely to develop diabetes, which warrants further evidence from a well-designed prospective study.

A significant body of evidence among T2DM patients suggests that increased age, gender (female), obesity, and thyroid peroxidase antibody positivity are associated with an increased risk of developing hypothyroidism [49, 56–58]. The prevalence of subclinical hypothyroidism is known to increase with age [10–12, 16–19]. Males and females have different propensities for thyroid dysfunction [59], and obesity has been found to be significantly related to hypothyroidism [60]. A meta-analysis of 36 studies confirmed a higher prevalence of subclinical hypothyroidism in females and T2DM patients over age 60 [43]. Furthermore, a cross-sectional observational study among 1508 adult T2DM patients in India found a significant increased risk of hypothyroidism in elderly patients with T2DM (> 65 years) with an OR of 4.2, and an overt difference between males and females (OR 4.82 vs. 2.60), and patients with obesity and without obesity (OR 2.56 vs. 3.11) [61]. However, our subgroup analyses found a consistent and positive association between hypothyroidism and risk T2DM regardless of sex (women ≥ 50% vs. women < 50%), age (< 60 years vs. ≥ 60 years) of participants in the studies, and outcomes (T2DM vs. T2DM & T1DM). The hypothyroidism causes a decrease in basal metabolic rate and thermogenesis and leading a weight increase [62]. As we have known, overweight/obesity is an overt risk of T2DM [63]. It is reasonable to deduce that the association between TSH and T2DM would be stronger among those with overweight or obesity. However, our study showed a conflict result that the association between high TSH and T2DM risk was non-significant among those with overweight/obesity. This might be caused by our BMI subgroups which were only according to the mean BMI levels of all participants since no identified study reported the results for the BMI subgroup and do not represent the exact overweight/obesity population. Similarly, a significant subgroup of medication use was found; however, the difference between those non-users and mixed users was non-significant. Few studies have explored the sex and age difference in association between thyroid function and T2DM, and those that have did not find any statistical significance [39]. Sex hormones, influenced by age, sex, and BMI status, may further play a complicated role in the T2DM and thyroid function [64]. The mixture of participants with different proportions of age, gender, and BMI groups and a limited number of relevant studies precluded us from evaluating the interaction of these factors.

Both hypothyroidism and hyperthyroidism are able to influence the metabolism of insulin and thus induce insulin resistance [39], suggesting a non-linear, possible U-shaped, relationship between thyroid function and diabetes. An L-shaped relationship between FT4 levels and metabolic syndrome incidence was observed in the prospective population-based Tehran Thyroid Study [65]. Our dose-response analysis of 9 studies depicted a J-shaped relationship with TSH and inverted-J shaped relationship with both FT3 and FT4 levels with T2DM, showing a relatively high risk of T2DM among those with elevated TSH levels and reduced FT3 and FT4 levels. The range of the thyroid function levels in most of the included studies was a semi-closed interval, with values only on one side of the reference range. We hypothesize that this might be a reason why we did not detect a U-shaped relationship. There appears to be a positive linear relationship between TSH and risk of T2DM even within the normal reference range according to a 7-year longitudinal study on 6235 euthyroid subjects without pre-existing T2DM [25]. These results suggest that lower levels of thyroid hormone might contribute to a higher risk of insulin resistance and diabetes [66, 67]. We assessed the influence of thyroid function on CVD among T2DM patients and did not find an association, though this was based on only 4 studies.

In this study, we conducted a comprehensive meta-analysis of prospective studies in order to evaluate the association between thyroid function and T2DM and CVD prognosis of T2DM patients. All studies included were sufficiently high-quality with an NOS score of ≥ 6 points and had large sample sizes (range: 556–91,120). In addition, a subgroup analysis stratified by prespecified factors and a sensitivity analysis was conducted to explore and evaluate the potential heterogeneity. Several limitations merit consideration. First, most of the studies included for evaluating the progress of T2DM were from China and more well-designed, large-scale, and long follow-up studies should be included in any future analyses. Second, only a few studies reported the glucose levels and evaluated the relations between thyroid function and glucose levels. Third, most studies did not distinguish between type 1 and type 2 diabetes. Additionally, only one study [35] evaluated thyroid peroxidase antibody titers. Finally, publication bias and residual confounders inherent in observational studies also merit further consideration.

## Conclusion

In summary, our meta-analysis demonstrated that thyroid dysfunction is associated with an increased risk of T2DM. There was no evidence that thyroid dysfunction was associated with CVD events and all-cause mortality in T2DM, though limited studies were available. Therefore, measuring TSH in patients with risk factors for diabetes may help further assess the risk for the development of T2DM.

## Supplementary Information

**Additional file 1. **

## Data Availability

All data generated or analyzed during this study are included in this published article.

## Abbreviations

CI: Confidence interval
CVD: Cardiovascular disease
HbA1c: Glycated hemoglobin
HR: Hazard ratio
NHANES III: Third National Health and Nutrition Examination Survey
NOS: Newcastle-Ottawa Scale
OR: Odds ratio
PRISMA: Preferred Reporting Items for Systematic Reviews and Meta-Analyses
RR: Relative risk
T2DM: Type 2 diabetes mellitus (T2DM)
T3: Triiodothyronine
T4: Thyroxine
TSH: Thyroid-stimulating hormone
