# Supplementary material for: Association between thyroid dysfunction and type 2 diabetes: a meta-analysis of prospective observational studies
Source: BMC Med. 2021 Oct 21;19:257. doi: 10.1186/s12916-021-02121-2 (PMC8529738; doi:10.1186/s12916-021-02121-2)
Supplement: Supplementary file 1 — Additional file 1. [file 12916_2021_2121_MOESM1_ESM.docx]

1. **Strategy for PubMed, DATE: 2021/5/1**
2. **Thyroid disfunction:** (“subclinical hyperthyroidism”[Title/Abstract] OR “subclinical hypothyroidism”[Title/Abstract] OR “subclinical thyroid dysfunction”[Title/Abstract] OR “Thyroid Function Tests”[Title/Abstract] OR “Thyroid Function Tests”[MeSH Terms] OR “thyroid hormone”[Title/Abstract] OR “thyroid hormone”[MeSH Terms] OR “thyrotropin”[Title/Abstract] OR “thyrotropin”[MeSH Terms])
3. **T2DM:** (“prediabetes”[Title/Abstract] OR “prediabetes”[MeSH Terms] OR “glucose intolerances”[Title/Abstract] OR “glucose intolerances”[MeSH Terms] OR “Oral Glucose Tolerance test”[Title/Abstract] OR “Oral Glucose Tolerance test”[MeSH Terms] OR “hemoglobin a1c”[Title/Abstract] OR “hemoglobin a1c”[MeSH Terms] OR “insulin”[Title/Abstract] OR “insulin”[MeSH Terms] OR “insulin resistance”[Title/Abstract] OR “insulin resistance”[MeSH Terms])
4. **(i AND ii)** Filters: **Humans; English; Adult: 19+ years**
5. **Strategy for Embase, DATE: 2021/5/1**
6. **Thyroid disfunction:** ('subclinical hyperthyroidism':ab,ti OR 'subclinical hypothyroidism':ab,ti OR 'subclinical thyroid dysfunction':ab,ti OR 'thyroid function tests':ab,ti OR 'thyroid function tests'/exp/mj OR 'thyroid function tests'/mj OR 'thyrotropin':ab,ti OR 'thyrotropin'/exp/mj OR 'thyrotropin'/mj OR 'thyroid hormones':ab,ti OR 'thyroid hormones'/exp/mj OR 'thyroid hormones'/mj OR 'thyrotropin-releasing hormone':ab,ti OR 'thyrotropin-releasing hormone'/exp/mj OR 'thyrotropin-releasing hormone'/mj)
7. **T2DM:** ('prediabetic state':ab,ti OR 'prediabetic state'/exp/mj OR 'prediabetic state'/mj OR 'glucose intolerance':ab,ti OR 'glucose intolerance'/exp/mj OR 'glucose intolerance'/mj OR 'glucose tolerance test':ab,ti OR 'glucose tolerance test'/exp/mj OR 'glucose tolerance test'/mj OR 'glycated hemoglobin a':ab,ti OR 'glycated hemoglobin a'/exp/mj OR 'glycated hemoglobin a'/mj OR 'insulin':ab,ti OR 'insulin'/exp/mj OR 'insulin'/mj OR 'insulin resistance':ab,ti OR 'insulin resistance'/exp/mj OR 'insulin resistance'/mj)
8. **(i AND ii)** Filters: **Humans; English; Adult: 19+ years**
